# Design and optimization of metformin hydrophobic ion pairs for efficient encapsulation in polymeric drug carriers

**DOI:** 10.1038/s41598-022-09384-6

**Published:** 2022-04-06

**Authors:** Sara I. Abd-El Hafeez, Nermin E. Eleraky, Ehsan Hafez, Sara A. Abouelmagd

**Affiliations:** 1 Department of Pharmaceutics, Faculty of Pharmacy, Assiut University, Assiut, Egypt; 2 Institute for Drug Development and Innovation Research, Assiut University, Assiut, Egypt

**Keywords:** Drug development, Biomedical engineering, Drug delivery, Biomaterials, Molecular self-assembly, Nanoparticles, Nanoscale materials, Structural properties

## Abstract

Loading small molecular weight hydrophilic drugs into polymeric carriers is a challenging task. Metformin hydrochloride (MET) is a highly soluble oral antidiabetic drug of small size and high cationic charge. Hydrophobic ion pairing (HIP) is an approach for reversible modulation of solubility and hydrophilicity of water-soluble drugs via complexation with oppositely charged molecules. Herein, we prepared MET ion pairs and carefully studied and characterized MET interaction with different ligands, with the aim of increasing MET lipophilicity and loading efficiency. HIP was successful using three hydrophilic anionic ligands; sodium dodecyl sulphate (SDS) Carbopol (CB) and tannic acid (TA). Electrostatic interaction and hydrogen bonding drove the complexation per spectroscopic and thermal studies. Complexation efficiency depended on ligand type and charge ratio. While complexes had varying interaction strengths, the excessive stability of TA/MET resulted in unfavorable poor MET dissociation. Notably, HIP imparted a 450 and tenfold lipophilicity increase for SDS/MET and CB/MET, respectively. The latter showed favorable controlled, yet complete release of MET at pH 6.8 and was loaded into alginate beads. Complex bulkiness and decreased lipophilicity resulted in a dramatic 88% increase of MET loading, demonstrating the success of HIP as a simple, efficient and applicable approach for modulating drug’s properties.

## Introduction

Metformin hydrochloride (MET) is a cationic biguanide drug widely used for chronic management of type-2 diabetes. However, its high-dose (500-mg), suboptimal bioavailability (40–60%), and short half-life (2–6 h)^1,2^ demand repeated oral dosing^3–5^. Frequent dosing, on top of MET’s gastrointestinal side effects, adversely affects patient compliance and therapy success. This challenge can be addressed through sustained-release formulations, where drug is given less frequently. Nevertheless, MET’s low molecular weight (165.65-g/mol), hydrophilic nature, and high-dose make its loading into oral polymeric carriers challenging.

Hydrophobic ion pairing (HIP) has emerged as an efficient approach to modify unfavorable physicochemical qualities of ionizable hydrophilic drugs. In HIP, ionizable water-soluble drugs are stoichiometrically complexed with counter ions driven by electrostatic interaction, resulting in a less soluble uncharged ion pair complexes^6–9^. HIP is driven by non-covalent interactions; thus, the complex can rapidly dissociate in the presence of ions to the native drug. This approach has been used to increase encapsulation efficiency of various biomolecules, peptides, peptide antibiotics^7,10^, anticancer drugs^11^, and other water-soluble drugs^12,13^.

Here, HIP is examined as a strategy to modulate the MET properties and enable its efficient loading into a standard oral polymeric carrier (alginate beads). Such approach offers several advantages; (1) ion-paired MET complex will be less hydrophilic and bulkier than free MET, hence easier to load into beads, and (2) a sufficiently stable complex will sustain MET release from the beads. Alginate beads were selected as polymeric carrier because of their biocompatibility and extensive use as an oral carrier. Moreover, alginate acid-resistance would protect complexed MET from gastric pH, then release it in the intestine^14^. Such ion-paired complex-in-beads would result in optimal pharmacokinetics, milder side effects less frequent dosing, and thus, higher patient compliance and therapy success.

We designed and optimized MET ion-paired complexes, testing five structurally diverse anionic ligands; Carbopol® 940 (CB), sodium dodecyl sulfate (SDS), tannic acid (TA), sodium deoxycholate (SDC), and low-methoxy low-amidated pectin (LMP). The interaction of each ligand with MET and the resulting ion pair, were carefully examined with the aims of (1) understanding how the two molecules interact at different ratios, (2) determining the strength of interaction and complex stability, and (3) evaluating how HIP modulated MET properties. A great discrepancy in ion pair properties and performance per ligand structure and nature of interaction with MET were observed. The most optimum complex was used to form MET-loaded alginate beads. Our complex-loaded beads were shown to have a dramatically higher loading efficiency than those loaded with free MET or polymer blend.

## Results and discussion

### Screening of anionic ligands

**Figure 1:**
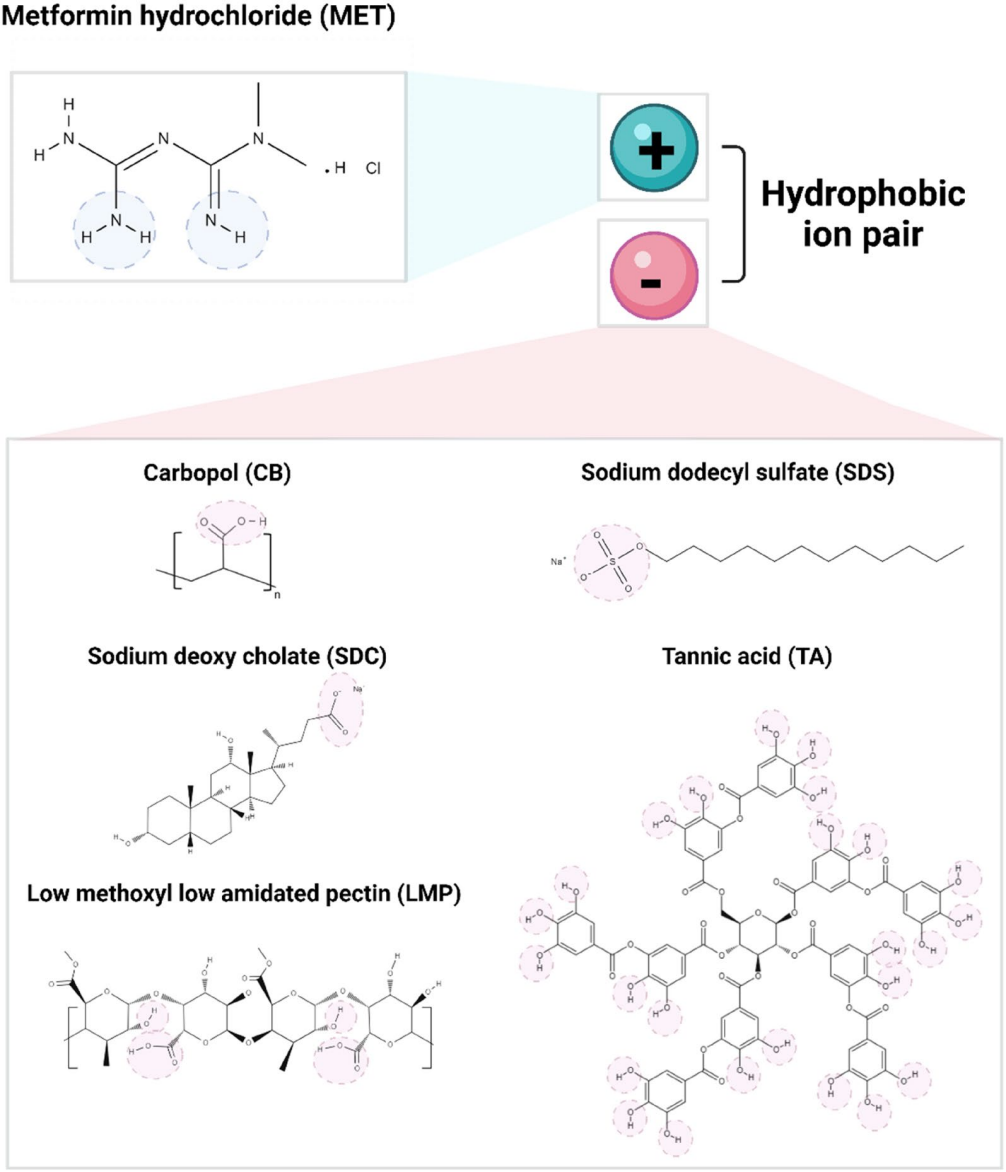
Chemical structures of metformin hydrochloride (MET) and different anionic ligands screened for hydrophobic ion-pairing (HIP). Charged moieties are indicated with circles.

MET is a cationic drug with two guanidine moieties^15,16^. It has two *pK*_*a*_ values (2.8 and 11.5) thus it exists in a neutral, mono- or di-protonated form based on medium pH (Fig. 1); diprotonated at pH < 2.8, monoprotonated at pH 2.8–11.5, and neutral at pH > 11.5^15,17^. Five different ligands were screened for HIP with MET: CB, SDS, TA, SDC, and LMP (Fig. 1). While all five are water-soluble and carry a negative charge (s), they have various molecular weights, charge densities, and charge profiles; CB and LMP are large molecular weight-polymers with several ionizable groups, TA is a polyphenolic compound rich in hydroxyl groups, SDS is a surfactant with a single ionizable group, and SDC is a bile salt that carries a carboxylic acid group.

**Figure 2:**
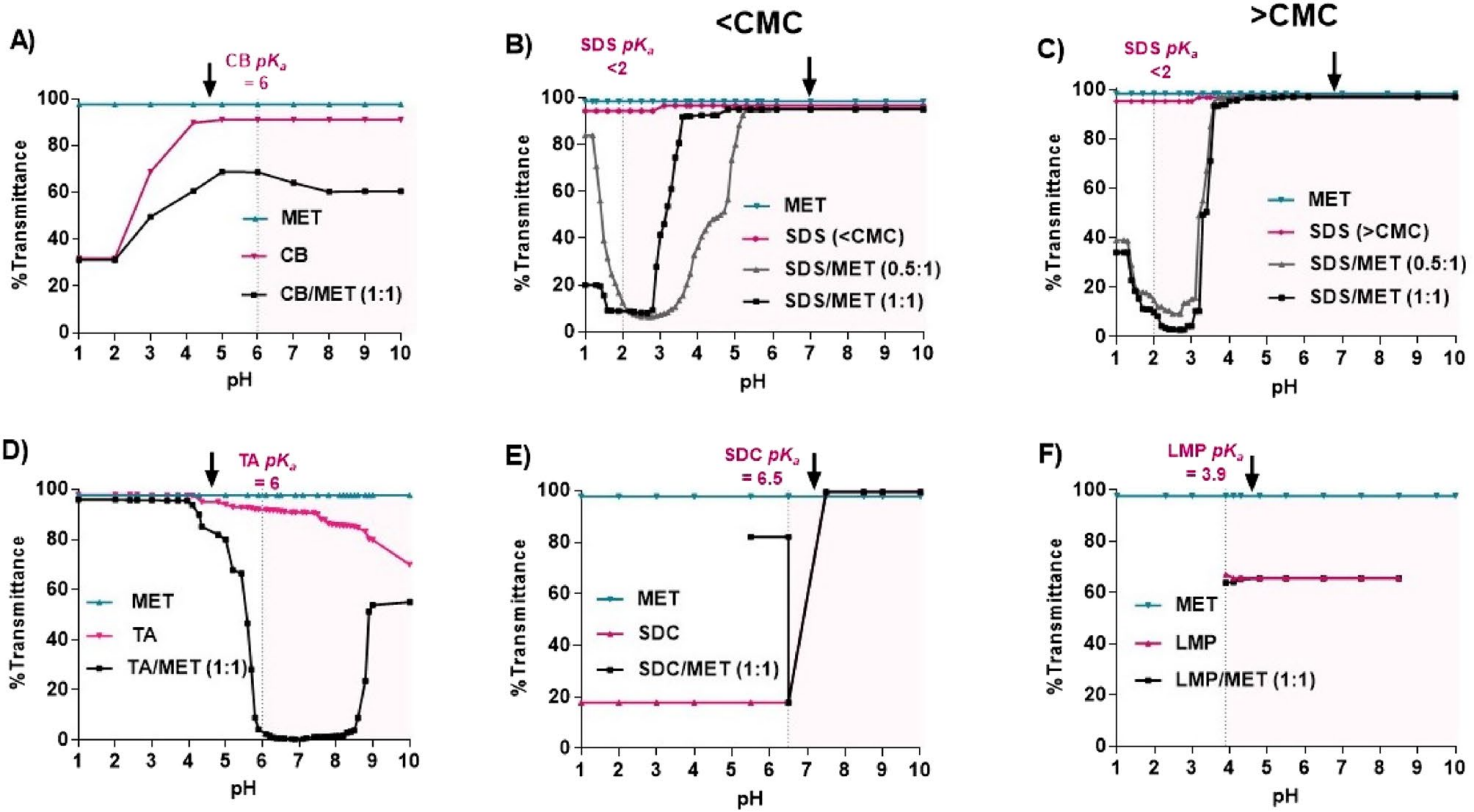
%Transmittance change versus pH for metformin hydrochloride (MET) with different ligands. (**A**) Carbopol (CB), (**B**) Sodium dodecyl sulfate (SDS) below critical micelle concentration (CMC), (**C**) SDS above CMC, (**D**) Tannic acid (TA), (**E**) Sodium deoxycholate (SDC), and (**F**) Low molecular weight pectin (LMP), compared to free MET and free ligand solutions. Black arrows show starting pH of ligand/MET mixture before pH adjustment. The pink plot area shows the pH range where both MET and ligand are ionized.

Ligands were screened for hydrophobic ion pair formation with MET by mixing the two at equal charge ratios (Supplementary Table 1) and varying solution pH. Turbidity of the solution was observed and compared to that of individual ligand solutions; transmittance (%T) reduction was interpreted as complex formation. Free MET solution %T was unaffected by pH alteration (Fig. 2). MET/ligand complexation was expected to occur at a pH range where both are ionized, thus varied for each ligand per its *pK*_*a*_.

#### CB/MET complex

CB is a crosslinked polyacrylic acid polymer with a molecular weight of 104,400-g/mole. It is rich in carboxylic acid groups with a *pK*_*a*_ of 6.0. Therefore, ionic interaction can occur between the positively charged amino group of MET and negatively charged carboxylate group of CB at pH range of 6–10, as MET and CB are ionized^18,19^. CB/MET suspension (1:1 charge ratio) exhibited lower %T levels at pH 3–10, showing interaction and formation of hydrophobic ion-paired complex, Fig. 2A. At pH < 2, CB precipitates; hence, %T reduced the most for CB and CB/MET samples at that range^20^.

#### SDS/MET complex

SDS is a surfactant with a negatively charged sulfate head group (*pK*_*a*_ < 2)^17^. SDS was previously reported to slow down MET release from tablets at pH 2 due to its interaction with MET at low pH level^17^. In our study, SDS solution was prepared at concentrations below and above CMC (0.0029 and 0.0061 M, respectively) to inspect the effect of micelle formation on the association between SDS and MET. SDS solution CMC was verified using conductimetry (Supplementary Fig. 1). Since SDS is charged at pH > 2 and MET would be either mono-or diprotonated at pH < 2.8 and higher, respectively. Two charge ratios of SDS/MET (0.5:1 and 1:1) were used at each condition. Free SDS solution was unaffected by pH change (Fig. 2B,C). Regardless of concentration used, SDS/MET solution remained clear at pH higher than 6.1, possibly because of the formation of nano complexes undetected through solution transmittance. Upon lowering the pH, %T gradually reduced. For all SDS/MET solutions, a sharp drop in %T occurred at pH ~ 3.6, indicating complex formation, except for 0.5:1 ratio prepared at a concentration below CMC; %T reduced at pH 5.3. The optimum pH for SDS/MET complex formation was determined to be pH 2.7 for further studies.

#### TA/MET complex

TA is a highly anionic polyphenolic compound containing ten phenol-rich gallic acid units with a *pK*_*a*_ ~ 6. TA is ionized at pH > 4 and completely ionizes at pH 8. As indicated in Fig. 2D, %T of TA/MET remained high at acidic pH (clear solution). %T drastically reduced with pH increase, reaching its lowest value at pH 5.9, where a buff-colored precipitate was formed, showing strong interaction between negatively charged TA and MET. %T increased again at pH 8, likely due to the decrease in hydrogen bonding^21^. Thus, the optimum pH for TA/MET complexation was determined to be 5.9. For free TA solution, %T showed a gradual reduction at pH > 5.9 due to reported TA instability and oxidation at such pH range, causing aggregation and solution color change^10^.

#### SDC/MET complex

SDC is an anionic bile salt with a *pK*_*a*_ of 6.5. Complex formation with MET was predicted to occur because of the interaction between MET and its hydrogen donor sites (carboxylic and two hydroxyl groups)^22^. However, %T-pH profile of SDC/MET was not different from SDC alone at pH > 6.5 (Fig. 2E). At pH 6.5, SDC/MET solution’s %T sharply reduced reaching 17.8%, then by lowering the pH, the solution gelled, increasing %T to 82%. Gelling of SDC was attributed to the presence of chloride ions used to adjust pH, leaving no room for the HIP. While successful HIP of SDC with insulin was previously reported^22^, gelling observed in our case with SDC/MET was in accordance with Sun et al*.* work, where SDC hydrogels formed in the presence of chloride salts^23^. Therefore, SDC was excluded from the study.

#### LMP/MET complex

LMP is an anionic polysaccharide, where each monomer contains a carboxylic group imparting an anionic charge (*pK*_*a*_ = 3.9). LMP was previously reported to form nanoparticles with cationic debranched starch driven by electrostatic interaction^24^. When screened for interaction with MET, %T profile was no different from that of free LMP (Fig. 2F). LMP/MET formed a viscous solution upon mixing with a starting pH of 4.5 and a reduced %T. Upon pH decrease to 3.9, solution viscosity increased, while increasing the pH did not cause any change in %T. Thus, LMP was excluded from the study as an ion pairing ligand.

### Nature of interaction between MET and ligands

Three ligands, CB, SDS, and TA, were selected for further studies of MET HIP. While %T-pH profiles validate the interaction between MET and the three ligands, it is essential to investigate: (1) mode of the interaction and (2) whether such interaction exists at various ligand/MET ratios. The interaction within the pair is likely a contribution of ionic and non-ionic interactions, such as hydrogen bonding and hydrophobic interactions^25^. The interaction strength should be high enough to drive a stable pair assembly yet allow the drug to be free to exert its physiological actions. Excessive interactions could cause an indissociable complex that cannot liberate the drug upon requirement. Complexation was performed by pH adjustment to a level where drug and ligand are ionized, and thermal and spectroscopic methods were used for the study.

#### Differential scanning calorimetry (DSC)

**Figure 3:**
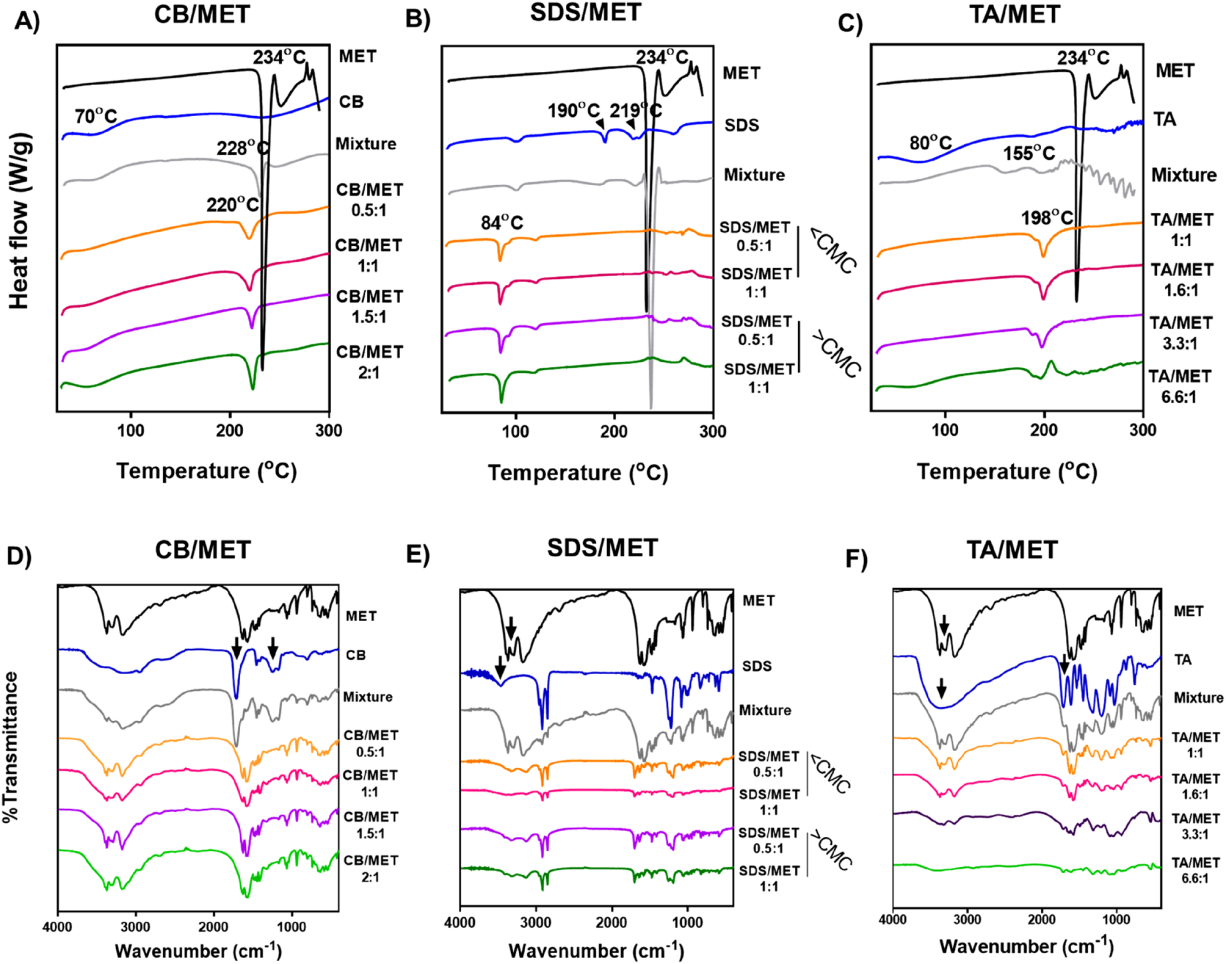
Differential scanning calorimetry (DSC) thermograms (**A**–**C**) and FTIR spectra (**D**–**F**) of metformin hydrochloride (MET) complexes. Complexes prepared using Carbopol (CB), sodium dodecyl sulfate (SDS), and tannic acid (TA) prepared at different charge ratios. Spectra are compared to that of free MET and ligands (CMC; critical micelle concentration, Mixture; the physical mixture of MET and ligand at 1:1 charge ratio).

DSC thermograms of MET, ligand, physical mixture, and freeze-dried ligand/MET complexes are presented in Fig. 3A–C. MET indicated a characteristic sharp endothermic peak at 234 °C because of melting, followed by an exothermic decomposition peak at 250 °C^15,26^. As reported, the CB thermogram (Fig. 3A) showed a peak at 70 °C due to glass transition and another at 256 °C due to decomposition^27^. For the CB/MET physical mixture, MET’s 234 °C peak was shifted to 228 °C and appeared with a lower intensity. However, the peak further shifted to 220 °C with CB/MET complexes of various charge ratios, showing interaction and complex formation.

For SDS/MET thermograms, SDS showed characteristic endothermic peaks at 101, 190, 219.25, and 257.56 °C^28^, where the 219.25 °C peak is attributed to SDS melting (Fig. 3B). While no signs of interaction appeared in SDS/MET physical mixture thermogram, 190 and 234 °C peaks were absent from the thermogram of all SDS/MET complexes, confirming specific interaction between MET and SDS in the complex. Additionally, thermograms of all complexes showed a new endothermic peak at 84 °C with the same intensity across samples showing concentration-independent eutectic mixture formation (above or below CMC).

Finally, for TA samples (Fig. 3C), TA itself showed an endothermic peak at 80 °C due to dehydration and decomposition, followed by reported combustion from 220 to 300 °C^29^. A new endothermic peak appeared at 155 °C with the mixture, showing some form of interaction. However, MET’s 234 °C peak disappeared, and a new peak appeared at 198 °C in a complex thermogram, indicating complex-specific interaction. Overall, the thermal analysis showed specific interactions between MET and tested ligands across various charge ratios, different from that seen with physical mixtures. The nature of these interactions and involved functional groups is further elucidated through Fourier-transformed Infra-Red (FTIR) spectroscopy.

#### Fourier-transformed Infra-Red (FTIR) spectroscopy

FTIR spectra were obtained for free MET, free ligands, ligand/MET physical mixtures, and freeze-dried complexes at different charge ratios (Fig. 3D–F). MET spectrum demonstrated characteristic sharp peaks at 3370, 3295.5, and 1559.8-cm^−1^ corresponding to NH stretching of the amine group (expected site of complexation), NH stretching of the imido group, and C=N stretching, respectively^15,30^.

For CB (Fig. 3D), characteristic peaks appear at 3177.4, 1714, 1270, 807, and 605-cm^−1^ corresponding to carboxylic O–H stretching, carboxylic C=O stretching, carbocyclic C–O stretching, =C–H alkenes bending, and –C=C–H alkyne bending, respectively^31^. Physical mixture indicated peaks of CB and MET, but CB/MET appeared differently; CB’s 1714 and 1270-cm^−1^ peaks disappeared for all ratios (arrows in Fig. 3D), possibly because of increased ionic interaction. Similar results were reported with SDC ion pairs with insulin; carboxylate group stretching peak of SDC at 1564.0-cm^−1^ disappeared, showing its interaction with amine group of insulin^22^. Contrary to predicted, strong hydrogen bonding was not observed in complex spectra, and MET peaks did not disappear or show reduced intensity, likely because of relatively loose CB/MET interactions.

For SDS/MET, Fig. 3E, free SDS spectrum indicated its characteristic peaks; a broad peak at 3465-cm^−1^, a forked peak at 2973-cm^−1^, and peaks at 2922, 1470, 2845, and 1470-cm^−1^, corresponding to hydrogen bonding, asymmetric CH_2_ stretching, symmetric CH_2_ stretching and asymmetric CH_3_ deformation, respectively^32^. Physical mixture indicated characteristic peaks of SDS and MET but with lower intensity because of dilution. Same as observed with CB/MET, complex samples appeared different from the mixture; SDS’s 3465-cm^−1^ peak and MET’s 3370-cm^−1^ NH stretching peak disappeared (arrows in Fig. 3E), while imido group NH stretching peak (3295.5-cm^−1^) reduced in intensity, especially at 1:1 (below CMC). This could be explained by ionic interaction and was observed in the remaining charge ratios. Similar behavior was reported with hydrophobic ion pairs of dioctyl sulfosuccinate and octreotide, where the vibration peaks were highly diminished and even disappeared due to the formation of the HIP complex^33^.

When it comes to TA/MET complex (Fig. 3F), the spectrum of TA showed characteristic broad peaks of phenolic O–H stretching appearing at 3670 to 2500 cm^−1^ attributable to intermolecular and intra-molecular hydrogen bonding. Also, O–H bending appeared at 1440 cm^−1^, and ester C=O appeared at 1720 cm^−1^^10,29^. Physical mixture showed TA and MET characteristic peaks with TA’s ester C=O stretching appearing as a shoulder. In TA/MET complex spectra, many changes were observed; (1) intensity of TA’s phenolic broad peak and C = O shoulder peak reduced, showing the availability of phenolic groups for hydrogen bonding with MET, and (2) intensity of MET sharp 3370-cm^−1^ peak and imido’s 3295.5-cm^−1^ peak reduced. The same pattern was observed with other complex ratios where TA and MET peaks intensity reduced with charge ratio increase. This pattern confirms predicted hydrogen bonding between TA’s phenolic OH and MET’s amine.

In summary, FTIR data is in accordance with thermal analysis; MET does interact with tested ligands in formed complexes at various ratios. CB/MET and SDS/MET complexes appear to be dominated by ionic interactions, while strong hydrogen bonding was noticed with TA/MET complex^34^.

### Characterization of MET/anionic ligands association complex

#### Complexation efficiency (CE%)

**Figure 4:**
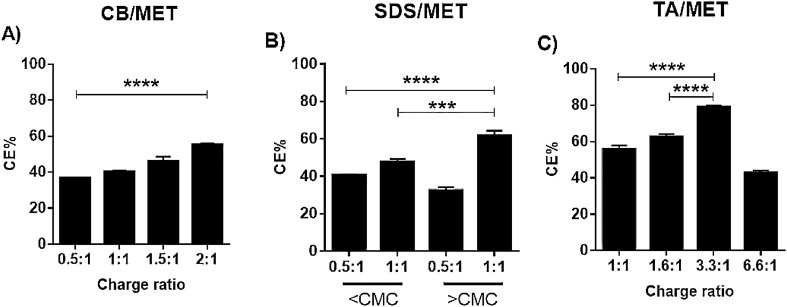
Complexation efficiency (CE%) of MET complexes. (**A**) Carbopol (CB), (**B**) sodium dodecyl sulfate (SDS), and (**C**) tannic acid (TA), prepared at various charge ratios (***p ≤ 0.001, ****p < 0.0001, One-way ANOVA, Tukey’s multiple comparisons test, CMC; critical micelle concentration).

After complex formation was verified and the mode of interaction was elucidated, the efficiency with which MET was complexed, CE%, was measured (Fig. 4). Mainly, the effects of ligand type and ligand to MET ratio were evaluated. Complexation is predicted to be driven by electrostatic interaction. However, other forces such as hydrogen bonding can contribute to the assembly thus, complexation should be studied at charge ratios lower and higher than 1:1. Overall, higher CE% is favorable as less drug is being lost during the complexation process.

For TA/MET complexes, only indirect method was used to determine CE%; it was not possible to free MET after complex formation because of extensive hydrogen bonding and high stability. Complex was indissociable and remained unaffected by heating or organic solvents. For all complexes, CE% was charge-ratio dependent. CE% increased upon increasing charge ratio, which was expected; more ligand is available to complex with MET, in non-stoichiometric hydrogen bonding contribution. This likely occurred with CB/MET and TA/MET; higher CE% were observed at ratios > 1:1. CE% attained a maximum of 55.6% at CB/MET 2:1 ratio, and 79% at TA/MET 3.3:1 ratio (Fig. 4A,C).

For SDS/MET (Fig. 4B), using SDS at > CMC increased CE%, attaining 61.89% at 1:1; micelles formation likely further stabilized the complexes or entrapped additional MET. Nevertheless, a higher charge ratio did not always translate to higher CE%; increasing TA/MET charge ratio from 3.3:1 to 6.6:1 causing a 36% reduction in CE%. This is consistent with reported binding properties of polyanionic ligands, such as DNA to polyethyleneimine, where stable complexes are formed at optimum nitrogen/phosphate ratio^35,36^. Similar findings were reported by Abdelhamid et al*.* for alendronate polyethyleneimine ion complexes^37^.

For further tests, the charge ratio of the highest CE% from each complex was used. CB/MET, SDS/MET, and TA/MET at 2:1, 1:1 (> CMC) and 3.3:1 having CE% of 55.5, 61.5 and 78%, respectively, were selected.

#### Particle size analysis and morphology

**Figure 5:**
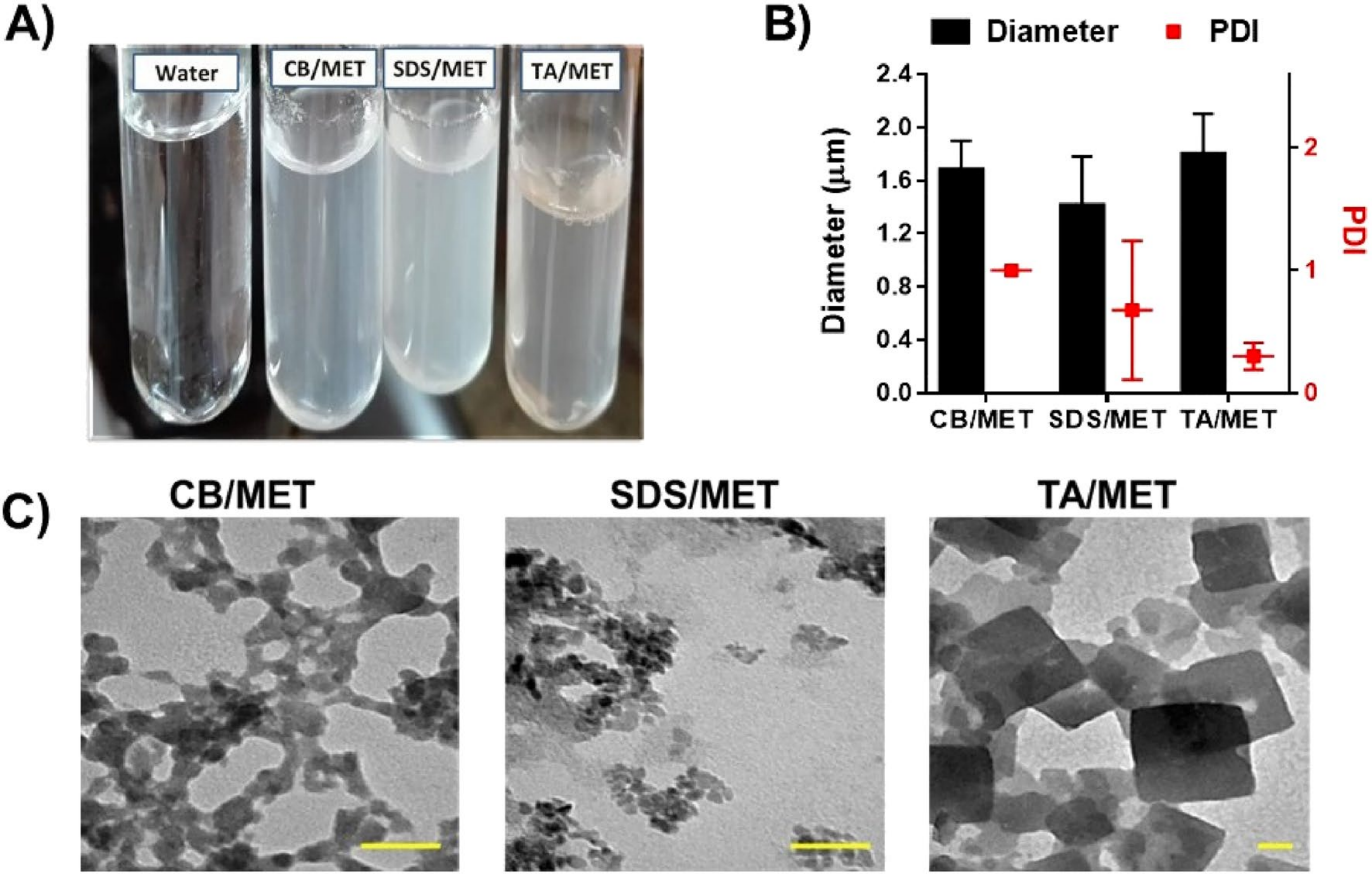
Morphology and particle size of different complexes. (**A**) Appearance of different complex suspensions, (**B**) particle size of complexes determined using dynamic light scattering, (**C**) Transmission electron microscopy (TEM) images of different MET complexes stained with 2% uranyl acetate (scale bar = 50 nm) (Charge ratios used; Carbopol/MET complex (CB/MET); 2:1, tannic acid/MET complex (TA/MET); 3.3: 1, and sodium dodecyl sulfate/MET complex (SDS/MET) 1: 1 (above critical micelle concentration); PDI; polydispersity index.

Visually, complex suspensions appeared turbid with a degree of translucency (Fig. 5A). Suspensions’ color was white except for TA/MET, which appeared buff-colored. Suspensions were analyzed through dynamic light scattering to determine their particle size. All suspensions were in the micron range (Fig. 5B). TA/MET complex had the largest particle size (1.801 ± 0.09 µm) followed by CB/MET (1.68 ± 0.22 µm), then SDS/MET (1.41 ± 0.37 µm). Polydispersity index (PDI) of CB/MET and SDS/MET was quite high showing polydisperse particles (Fig. 5B), while TA/MET was relatively more homogeneous with lower PDI. When imaged through Transmission electron microscopy (TEM) (Fig. 5C), complexes had different morphologies; CB/MET appeared as patches of crosslinked particles, while SDS/MET formed small, aggregated structures. Alternatively, TA/MET appeared as large cubic crystals, suggesting an ordered crystalline structure and explaining its poor dissociation. Compared to dynamic light scattering analysis, complexes appeared smaller upon imaging. This is because of different experimental conditions; TEM is done after the suspension is dried, while dynamic light scattering analysis is performed for hydrated and more aggregated particles. Overall, complexes’ size range enables easy collection through centrifugation and is suitable for further loading into polymeric carriers.

#### Partition coefficient

HIP's main aim is to increase the drug’s hydrophobicity facilitating its loading and delivery. The hydrophobicity of each MET complex, was measured by examining their partitioning between aqueous and organic phases. In counter ions absence, the partition coefficient of MET ranged from 0.059 to 0.064 at pH 8 and 2.7, respectively. HIP greatly increased MET’s hydrophobicity. For SDS/MET (1:1), *K*_*p*_ was equal to 29.1 ± 0.8, indicating a tremendous 450-fold increase in MET hydrophobicity, Supplementary Table 2. This is justified by the SDS surfactant effect previously reported for SDS-peptide complexes, causing a 200-fold hydrophobicity increase^38^. For CB/MET, a decent 10-folds increase in hydrophobicity was observed (*K*_*p*_ = 0.575 ± 0.079), Supplementary Table 2. TA/MET’s *K*_*p*_ was not measurable due to its poor dissociation. Nevertheless, MET hydrophobicity improvement via HIP with CB and SDS is appropriate for MET encapsulation into drug carriers and may modulate its permeability across biological barriers^33,39–41^.

#### Complex stability

From all results obtained, it is expected that TA/MET is the strongest complex. Stability is a crucial issue to examine when developing hydrophobic ion pairs; stability has to be optimized to ensure modulation of the drug’s lipophilicity yet allowing drug release. Conventional mathematical conversions using experimentally determined binding data were applied to evaluate complexes' stability. Following the standard addition (molar ratio) method (Supplementary Fig. 2A), the absorbance of the formed ligand/MET complex was plotted against the molar ratio [ligand]/[MET]. For SDS/MET, Job’s continuous variation method was applied (Supplementary Fig. 2B). Job’s method is a graphical representation of the absorbance of the formed complex versus ligand mole fraction. Job’s plot indicates enhanced absorbance of complexed drug with increasing ligand mole fraction untill it reaches a maximum value, after which further increase in ligand mole fraction, results in absorbance decreases.

**Figure 6:**
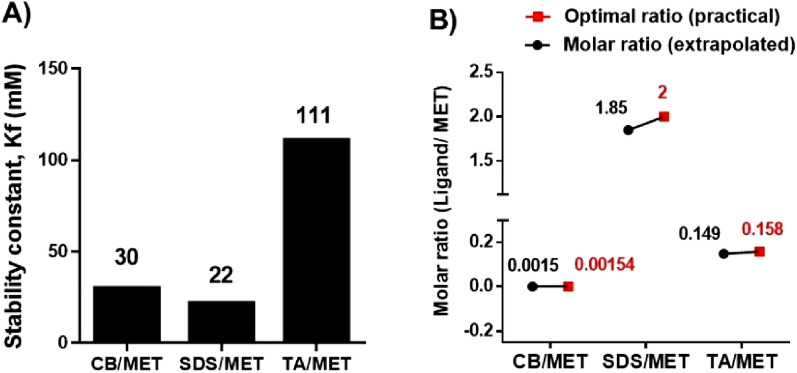
Stability constants and stoichiometric ratios of different MET complexes. (**A**) Estimated stability constants K_f_, and (**B**) stoichiometric ratios for Carbopol/metformin (CB/MET), Sodium dodecyl sulfate/MET (SDS/MET) and tannic acid /MET (TA/MET) complexes.

Comparing calculated *K*_*f*_ values determined for complexes, TA/MET was indeed a highly stable complex with a *K*_*f*_ of 111-mM (Fig. 6A). Previous reports that examined TA-lysozyme interaction indicated strong TA complexation mediated by non-covalent interaction^42^. In comparison, CB/MET and SDS/MET had much lower stability constants of 30 and 22-mM, respectively, meaning that SDS/MET is the weakest complex.

When examining the extrapolated stoichiometric molar ratios graphically obtained from the plots (Fig. 6, Supplementary Fig. 2), plot-determined ratios were closely related to the optimal molar ratios obtained experimentally. These latter ratios are ones determined to have the highest MET CE% (Fig. 4, Supplementary Table 1). The agreement between the two sets of values verifies that high CE% was a result of optimal interaction between MET and ligand.

#### In-vitro release of MET from complexes

Knowing how stable each complex is, it was essential to evaluate how such stability translates to drug release in various media encountered after oral administration (gastric pH of 1.2 and intestinal pH of 6.8). TA/MET is the most stable complex, and SDS/MET is the least stable with CB/MET in between.

**Figure 7:**
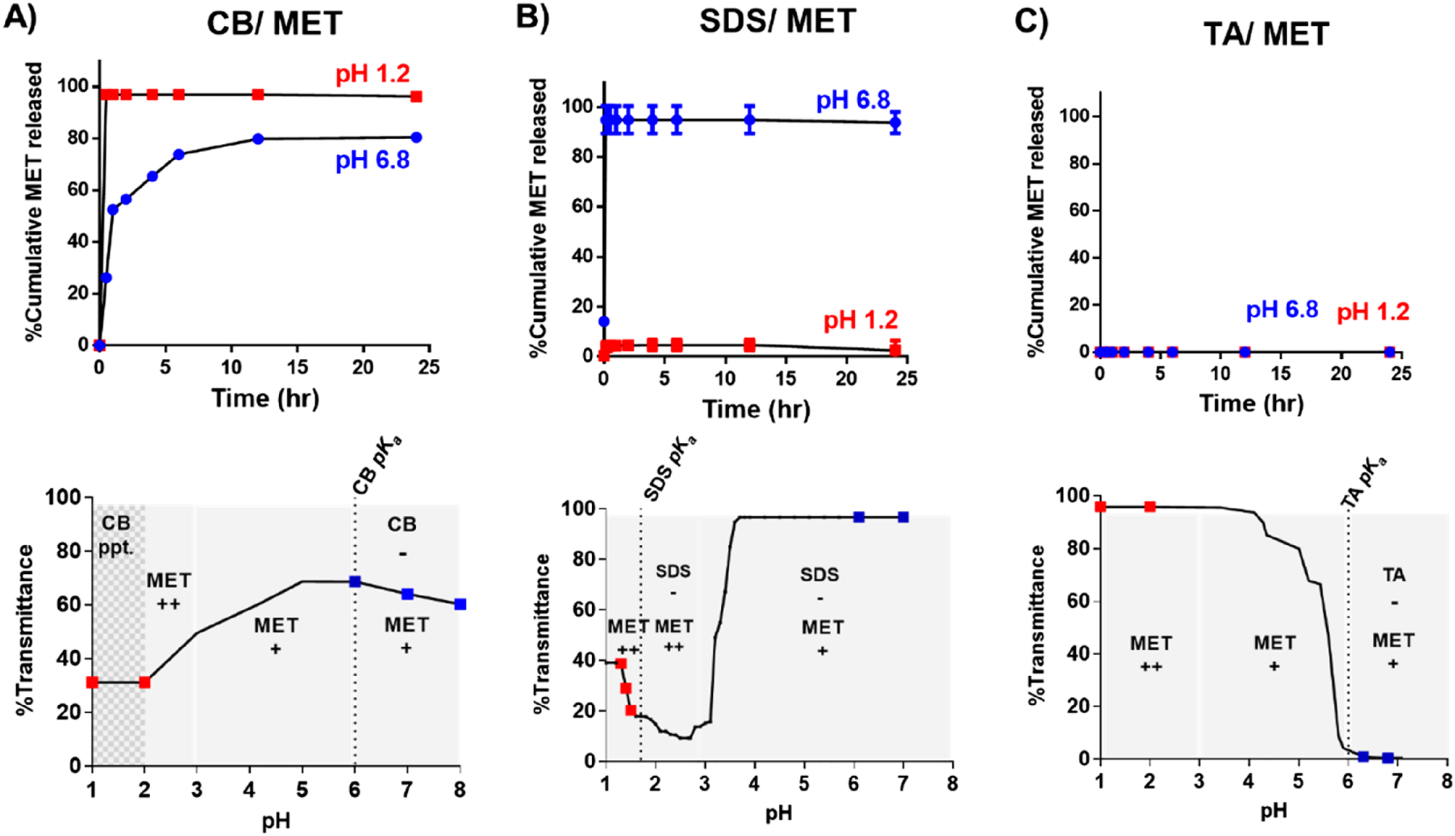
In-vitro release of metformin hydrochloride (MET) from complexes. (**A**) Carbopol/MET (CB/MET), (**B**) sodium dodecyl sulphate /MET (SDS/MET), and (**C**) tannic acid /MET (TA/MET) complexes of charge ratios 2:1, 1:1 (> CMC) and 3.3:1, respectively, and corresponding %transmittance-pH profiles of the three complexes with background indicating the ionization state of MET and ligands (release media; 0.2-M phosphate buffer of pH 6.8, and 0.1 N HCl).

*In-vitro* release profiles of the three complexes were pH-and stability-dependent (Fig. 7A–C). Each complex exhibited a distinct release pattern corresponding to MET-ligand association. CB/MET complex completely dissociated within 0.5 h at pH 1.2 while appearing stable at pH 6.8 (Fig. 7A); only 56% of MET was released in the first two hours. This discrepancy is explained by CB/MET %T-pH profile and CB’s *pK*_*a*_ of 6 (Fig. 7A); at pH 1.2, CB loses its charge and precipitates, releasing MET into the solution. However, at pH 6.8, CB and MET are charged, and complex slowly releases MET.

In contrast, SDS/MET association complex exhibited an opposite trend (Fig. 7B); only 2.5% of MET was released after 24 h at pH 1.2, but MET was released entirely within a few minutes at pH 6.8. From its %T-pH profile (Fig. 7B), the complex is stable at pH 1.2% as MET is diprotonated and SDS’s sulfate is charged (*pK*_*a*_ < 2), while monoprotonated MET at pH 6.8 is not charged enough to maintain complex stability resulting in MET burst release^17^.

On the other hand, from TA/MET’s %T-pH profile, its complete dissociation was expected at pH 1.2 as TA is unionized. However, it did not release any MET, at either pH level, for the entire 24 h (Fig. 7C). This was predicted by earlier experiments where TA/MET appeared in a cubic crystalline form under TEM (Fig. 5C), which showed strong hydrogen bonding (Fig. 3C) and had a 111-mM stability constant (Fig. 6A).

Finally, revisiting the aim of this work, the intention is to increase MET lipophilicity, thus (1) enabling its efficient loading into polymeric carriers (alginate beads), and (2) sustaining its release upon oral administration. Hence, optimum MET ion pair should abide by certain criteria; it should: (1) increase MET’s partition coefficient, (2) be stable enough to withstand loading, (3) sustain MET release at intestinal pH, and (4) achieve complete dissociation and MET release. Based on these criteria, TA/MET was excluded; the complex was indissociable (Fig. 7C). Comparing CB/MET to SDS/MET, they had comparable stability (30 and 22-mM, respectively) (Fig. 6A). However, their lipophilicities and release profiles varied. First, SDS/MET was far more lipophilic than CB/MET (Supplementary Table 2). Such lipophilicity increase (~ 455 fold) can adversely affect complex loading into alginate beads, as complex would tend to self-aggregate rather than be encapsulated into the beads. Second, SDS/MET instantaneously dissociated at pH 6.8, while CB/MET slowly released MET (Fig. 7A,B). Notably, dissociation of CB/MET at pH 1.2 would not compromise controlled MET release per oral delivery, as alginate beads will form a dense matrix at acidic pH, protecting the complex and preventing water penetration^43^. Therefore, CB/MET was selected as the optimum MET ion pair.

### Encapsulation of CB/MET complex into calcium alginate beads

**Figure 8:**
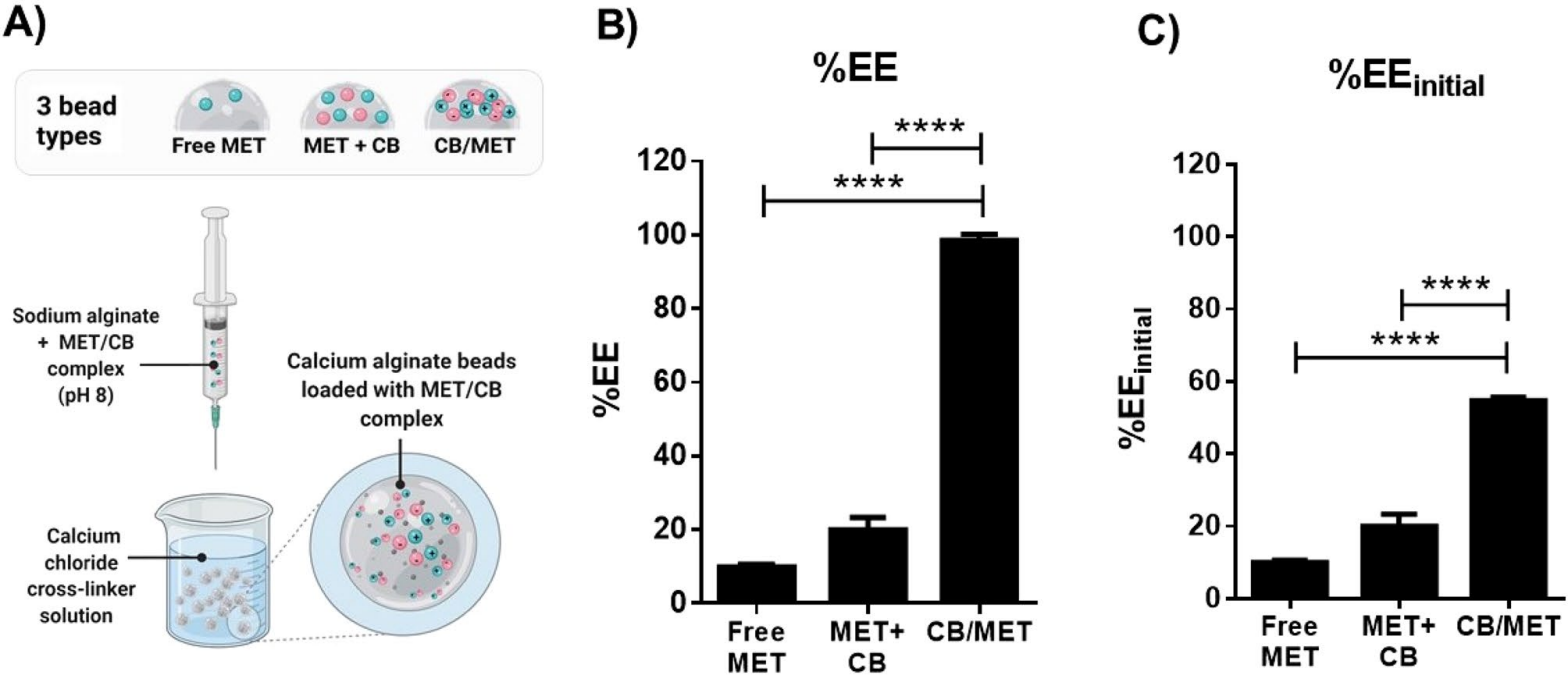
Preparation of calcium alginate beads and metformin hydrochloride (MET) encapsulation efficiency (EE). (**A**) Diagram indicating ionotropic gelation method used to prepare calcium alginate beads and bead types prepared. (**B**) Effect of the form used of MET on the EE into alginate beads. %EE was calculated based on the amount of complexed MET (CB/MET) added, and (**C**) %EE was calculated based on initial MET used for complexation (%EE _initial_) (MET + CB; a mixture of CB and MET (MET + CB) at amounts equivalent to that of CB/MET, Feed ratio of MET and sodium alginate concentration were 1:20 MET/alginate, and 5%w/v, respectively, ****p < 0.0001, One-way ANOVA, Tukey’s multiple comparisons test).

In the literature, many attempts were reported to enhance drug loading into alginate beads and modify their release profile. Among these, polymer blending was frequently used; a polymer such as CB would be incorporated into the alginate matrix to form a polymer blend that can better entrap loaded drug^44^. Therefore, in our study, three different types of beads were incorporated (Fig. 8A); (1) beads loaded with the free drug (MET), (2) beads loaded with CB/MET complex, and (3) polymer blended beads containing a mixture of CB and MET (no complex formation step, but equivalent amounts). Beads were prepared through ionic gelation of sodium alginate in calcium chloride solution (Fig. 8A).

The three types of alginate beads formed were white in color, spherical in shape, and 2.59–3.39-mm in diameter. Figure 8B shows the beads EE%. In comparison to free MET, CB/MET caused a dramatic increase in EE% (from 10.04 to 98.6%); almost all loaded complex was retained in the beads as they formed, while 90% of free MET escaped the beads were solidifying. This shows our ion-pairing approach's success in loading hydrophilic small molecular drugs into polymeric matrices. It is possible that CB presence as a polymer capable of hydrogen bonding may have helped retain MET within the alginate matrix as it solidified. However, beads prepared using the polymer blend (MET + CB) only increased EE% by 9.91% (Fig. 8B). This shows the minimal effect of CB blending, attributable to the higher viscosity of CB-alginate matrix and relatively denser beads^44^. Additionally, even when EE% is calculated considering the MET lost during the complexation procedure (EE%_initial_); CB/MET-loaded beads had far superior EE; 34% higher than MET + CB beads (Fig. 8C).

In summary, this study shows the applicability of the HIP approach for modulating the qualities of small hydrophilic drug molecules without affecting their native structures. Instead of chemical conjugation or complex formulation design, this approach is simple and green, involving no organic solvents, toxic reagents, or lengthy steps. CB is a common excipient used in the formulation of pharmaceutical products with a proven safety profile^45^. Favorably, HIP was optimized for a reversible yet stable association between CB and MET, pH-dependent. The outcomes of this study can be applied for hydrophilic molecules with similar difficulties in loading and delivery. Finally, our developed CB/MET-loaded calcium alginate beads are to be further optimized and studied in vitro and in vivo for their performance and safety as a sustained release platform for MET delivery.

## Materials and methods

### Materials

Metformin hydrochloride (MET) was a kind gift from Chemical Industries Development company (Cairo Egypt), SDS was bought from Research lab industries (Mumbai, India), Carbopol 940 (CB) and sodium alginate were bought from the general chemical, and pharmaceutical Company Ltd. (England), tannic acid (TA), sodium deoxycholate (SDC), and octanol were bought from Sigma- Aldrich (Germany), low-methoxy low-amidated pectin (LMP, 35% esterification, 15% amidation, molecular weight = 59 + 1.2 KDa) was bought from CP Kelco (Copenhagen, Denmark). The rest of the materials were bought from El-Nasr Pharmaceutical Chemicals Company (Cairo, Egypt).

### Ligand screening

Five anionic ligands (CB, LMP, SDC, SDS, and TA) were screened for the formation of MET/ligand association complexes. Complexation was indicated by a change in the solution’s % transmittance (%T) upon pH change. Optimum pH for complex formation was determined first, then adapted for further studies. Equal volumes of aqueous solutions of MET, and each ligand, were mixed at a 1:1 charge ratio, Supplementary Table 1. Ligand solution was added gradually to MET solution with stirring. The charge was calculated based on MET *pK*_*a*_ at screened pH range. Resulting Ligand/MET solutions adjusted with 0.1 M sodium hydroxide and/or 0.1 N hydrochloric acid. %T was recorded using a UV spectrophotometer at 520-nm (Jenway Ltd, Feslted, Dun mow, UK). Similarly, a change in %T for solutions containing only the ligand or MET was investigated. Optimum pH was defined as one that gave the lowest %T value in ligand/MET solution, indicating the formation of complexes^10^.

### Preparation of association complexes

Based on screening performed, association complexes of MET with CB, SDS, and TA were prepared at corresponding optimum pH levels. MET solution was gradually added to the ligand solution of equal volume accompanied with stirring. For CB/MET complex, thepH of MET and CB solutions was adjusted to eight (using 0.1-M sodium hydroxide) and the complex solution was stirred for 20 min CB/MET charge ratios of 0.5:1, 1:1, 1.5:1, and 2:1 were prepared^34,46^. For SDS/MET, complex aqueous solutions of SDS and MET were adjusted to pH 2.7 (optimum pH). SDS/MET charge ratios of 0.5:1 and 1:1 (below or above critical micelle concentration (CMC)) were used with 20 min stirring. SDS CMC was determined to explore the effect of micelle solvation on formed complex using conductimetry (Supplementary data). To achieve SDS concentration below and above CMC, the final SDS concentration in complex suspension was 0.087% and 0.174%, respectively. For TA/MET complex, phosphate buffer of pH 5.9 (200-mM) was used for complex preparation. TA/MET charge ratios of 1:1, 1.6:1, 3.3:1, and 6.6:1 were prepared by stirring for ten minutes.

### Characterization of complexes

#### Study of nature of interaction

DSC and FTIR spectroscopy were used to investigate the nature of interaction within complexes. Samples (4-mg) of MET powder, ligand, physical mixture, and freeze-dried complexes at different ratios were placed in aluminum pans sealed and heated at constant rate of 10 °C/min using computer-interfaced Shimadzu Calorimeter (Model DSC-50, Kyoto, Japan) in the range of 25–300 °C under a constant flow of nitrogen gas. For FTIR spectroscopy, samples were analyzed using Nicolet IS10 FTIR spectrometer (ThermoFischer, USA)^34^. Dry samples were pressed using potassium bromide-disks, and the infra-red spectra were recorded from 400–4000-cm^−1^ at room temperature.

#### Complexation efficiency

After complex formation, complexation efficiency (CE%) was determined either directly (for CB/MET, SDS/MET, and TA/MET complexes) or indirectly (TA/MET complex). In the direct method, the complex suspension was centrifuged to separate the complex from unentrapped MET. Samples were centrifuged for 15 min at 14,000 or 10,000-rpm for CB/MET and SDS/MET complexes, respectively. Then, obtained complexes were resuspended in a medium where the complex dissociates (0.1-M HCl for CB/MET and 0.2-M phosphate buffer (pH 6.8) for SDS/MET). Finally, samples were centrifuged a second time to separate precipitated ligand (SDS or CB) from supernatant containing freed MET. The supernatant was analyzed spectrophotometrically for MET content at ʎ_max_ = 235 nm. CE% was determined according to Eq. (1)^34,46^.

1 $$CE\% = \frac{MET\; amount\; from\; dissociated \;complex }{{Total\; MET\; amount}} \times 100$$

For TA/MET complex, CE% was analyzed directly and indirectly. Ninhydrin assay was used instead of UV spectrophotometry due to interference from TA. The complex suspension was analyzed for MET content twice. Firstly, MET content was determined in suspension as it is (without centrifugation, i.e., total MET content). Secondly, MET content was determined after centrifugation (6000-rpm, 30 min) and supernatant removal (i.e., complexed MET). CE% was calculated from Eq. (2). Alternatively, CE% was determined indirectly by analyzing the supernatant remaining after complex centrifugation, *i.e*., unentrapped MET. To avoid overestimation of CE% due to drug loss or adherence to tube. CE% was calculated for a standard MET solution (no TA was added) that underwent centrifugation and processed in parallel with a complex sample. Equation (3) was used for indirect CE% calculation.

2 $$CE\% \left( {direct} \right) = \frac{MET\;amount\;in\;collected\;complex\;after\;centrifugation}{{MET\;amount\;in\;sample\;without\;centrifugation}} \times 100$$

3 $$CE\% \left( {indirect} \right) = 100 - \left( {\frac{ MET\;contnet\;in\;supernatant\;after\;centrifugation }{{ 100\% \;sample\;without\;TA\;absorbance }} \times 100} \right)$$

For ninhydrin assay, first, ninhydrin reagent was prepared by dissolving 0.2-g ninhydrin and 0.03-g hydrindantin in 10-ml 3:1 dimethyl sulfoxide/sodium acetate buffer (pH 6.5)^10,47^. Second, complex samples (0.1-mL) were diluted to 0.5-ml with phosphate buffer (pH 5.9), and combined with 0.5-ml ninhydrin reagent. Samples were shaken, covered, placed in a boiling water bath for 20 min, then cooled and diluted with distilled water. The violet color developed was measured at λ_max_ = 567 nm spectrophotometrically.

#### Particle size analysis and morphology

Particle size and PDI of freshly prepared CB/MET, SDS/MET, and TA/MET complexes of selected charge ratios were measured through dynamic light scattering using Malvern Zetasizer ZS90 (UK). Samples were measured in triplicates at 25 °C. For morphology, complexes were imaged using TEM. The complex suspension was diluted with the same medium used for complex association (to ensure stability). A sample drop was placed onto a carbon-coated copper grid then stained with uranyl acetate solution (2% w/v). Excess staining was removed using filter paper, and the sample was allowed to air-dry. Then, the sample was imaged using the TEM (FEI Technai F20, USA)^48^.

#### Partition coefficient

Octanol/water partition coefficient was determined through shake flask method for ligand/MET complexes to determine the effect of the HIP on their hydrophobicity^49^. Partition coefficient of TA/MET was not determined because it was indissociable. Ten mg of free MET or equivalent in complex was added to 10-ml water (at pH 8 for CB/MET and pH 2.7 for SDS/MET), and 10-ml octanol in a flask. Flask was shaken for 72 h, then the phases were separated using a separating funnel and centrifuged at 6000 rpm to ensure complete separation. Next, the aqueous phase was analyzed for MET content. For CB/MET, the pH was adjusted to 2 using hydrochloric acid to release free MET from complex, then filtered through 0.45-µm filter disk, and diluted (tenfold) using phosphate buffer (pH 6.8). For SDS/MET, fivefold dilution was perfomed with phosphate buffer (pH 6.8), followed by filtration. MET concentration was determined spectrophotometrically at 235-nm.

Partition coefficient (*K*_*p*_) and fold increase in lipophilicity were calculated from Eq. (4–6)^19^. Partition coefficient was calculated by directly using measured MET content in water phase [MET]_W_, while MET amount in octanol [MET]_O_ was calculated by subtracting [MET]_W_ from total added drug amount [MET]_T_. Where *K*_*P*__*MET*_ _*complex*_ and *K*_*P*__*MET*_ are partition coefficients for complexed and free MET, respectively.

4 $$\left[ {MET} \right]_{O} = \left[ {MET} \right]_{T} - \left[ {MET} \right]_{W}$$

5 $$K_{P} = \left[ {MET} \right]_{O} / \left[ {MET} \right]_{W}$$

6 $$Folds\;increase\;in\;lipophilicity = K_{P MET\;complex} /K_{P MET}$$

#### Determination of stability constant and stoichiometric ratio

The stoichiometric ratios and stability constants of ligand/ MET complexes were estimated using either the standard addition or Job’s method of continuous variation. The latter was used for SDS/MET complex as its stoichiometry was expected to be in the range of 1:2–2:1, which is better assessed using Job’s method^50^. Different molar ratios of complexes were prepared in standard addition method at varied ligand (CB or TA) concentrations and fixed MET concentration. In Job’s method, equimolar solutions of MET and SDS were prepared, then mixed at varying ratios. The absorbance of complexed MET was determined for both methods, and data were plotted to determine the stoichiometric ratio and stability constants. Method and calculations are detailed in the supplementary information.

#### In-vitro release of drug from MET/ligand association complex

*In-vitro* release profiles of MET from freeze dried complexes were determined at pH levels resembling that of gastric fluid (0.1 M hydrochloric acid, pH 1.2) and intestinal fluid (0.2 M phosphate buffer, pH 6.8). Complexes equivalent to 10 mg MET were added to each medium (100 mL), incubated in a thermostatically controlled shaking water bath (Gallen Kamp, England) at 37 °C and 50 rpm. Samples were withdrawn at 1, 2, 4, 6, 12 and 24 h , and replaced with fresh medium. MET concentration was determined spectrophotometrically as mentioned before.

### Preparation of CB/MET- loaded alginate beads

The effect of MET HIP on its loading into calcium alginate beads was studied. Three types of beads were prepared; beads loaded with free MET, beads loaded with CB/MET complex, and beads loaded with a mixture of free MET and CB (in equivalent amounts to that in complex). For all, sodium alginate concentration and MET/sodium alginate ratio were kept at 5% and 1:20, respectively. Beads were prepared by ionotropic gelation method. MET or freeze-dried CB/MET of 2:1 charge ratio was dissolved or dispersed, respectively, in sodium alginate solution (pH 8). Then, MET-sodium alginate bubble-free solution was dropped using a 22-gauge needle into a 2.5% calcium chloride solution under gentle stirring (9–11 drops/minute) at 25-cm height from the solution (Fig. 8A). Volume ratio of sodium alginate to calcium chloride was kept at 2:3. Beads curation was allowed for ten min and beads were dried overnight at room temperature.

### Encapsulation efficiency of CB/MET in calcium alginate beads

For determination of encapsulation efficiency (EE%), a known weight of beads was crushed, dispersed in 0.2 M sodium citrate and sonicated for 30 min. MET concentration in filtered solution was measured at ʎ_max_ = 235 nm. To calculate the EE% of “complexed MET”, Eq. (7) was used. Alternatively, %EE was calculated from initial amount of MET used for complexation (EE%_initial_) from Eq. (8).

7 $$EE\% = \frac{Determined\;amount\;of\;complexed\;MET\;in\;beads}{{Amount\;of\;complexed\;MET\;used\;in\;beads\;preparation}} \times 100$$

8 $$EE \%_{initial} = \frac{Determined\;amount\;of\;complexed\;MET\;in\;beads}{{Amount\;of\;MET\;initially\;used\;for\;complexation}} \times 100$$

## Supplementary Information

Supplementary Information.
