# Supplementary material for: Design and optimization of metformin hydrophobic ion pairs for efficient encapsulation in polymeric drug carriers
Source: Sci Rep. 2022 Apr 6;12:5737. doi: 10.1038/s41598-022-09384-6 (PMC8986808; doi:10.1038/s41598-022-09384-6)
Supplement: Supplementary file 1 — Supplementary Information. [file 41598_2022_9384_MOESM1_ESM.docx]

**Supplementary information**

**Design and Optimization of metformin hydrophobic ion pairs for efficient encapsulation in polymeric drug carriers**

**Sara I. Abd-El Hafeez^1^, Nermin E. Eleraky^1^,** **Ehsan Hafez^1^_,_ Sara A. Abouelmagd^1,2*^**

*^1^Department of Pharmaceutics, Faculty of Pharmacy, Assiut University, Assiut, Egypt.*

*^2^Drug Research Center, Assiut University, Assiut, Egypt.*

*Corresponding author

Corresponding author:

**Dr. Sara A. Abouelmagd, PhD**

Associate Professor

Department of Pharmaceutics

Faculty of Pharmacy

Assiut University

Assiut, Egypt

E-mail: [sabouelm@aun.edu.eg](mailto:sabouelm@aun.edu.eg)

1. **Determination of critical micelle concentration (CMC) of sodium dodecyl sulphate (SDS)**
   1. **Methodology**

CMC value of SDS was verified conductometrically by using a conductimeter (Portable Conductivity Meter HI99300, Hanna company, Italy). Briefly, 50 ml of 0.04 M aqueous solution of SDS was prepared. Twenty five ml of acidified water (pH 2.7) were pippeted into a beaker. SDS solution was added to a beaker of purified water at 0.5 ml per addition followed by stirring. The conductivity was recorded, then plotted as a function of the SDS concentration. CMC was determined as the SDS concentration at the inflection point of plotted curve ^1^.

- 1. **Results**

Determination of CMC of SDS was done using a conductivity meter, the values of specific conductivities (k, µs/cm) were plotted against respective surfactant concentration. The inflection observed in the plot indicates the onset of aggregation and was set as CMC ^1^. The observed value of CMC of SDS in pH 2.7 was 0.0037 M (1.06 g/L) which is slightly lower than the value reported in literature (1.7- 2 g/L) ^1^, (**Supplementary Fig. 1**). Consequently, two concentrations were chosen for studying the binding interaction between SDS and MET. One below CMC (0.0029 M) to ensure the absence of micelle aggregation of SDS and one above CMC (0.0061 M) to test the effect of micelles solvate on the formed complex.


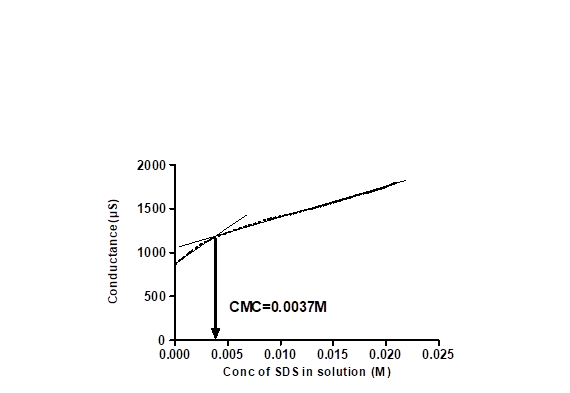


**Supplementary Figure 1:** Determination of critical micelle concentration (CMC) of sodium dodecyl sulphate (SDS) by conductimetric method.

1. **Determination of complex stability constant and stoichiometric ratio**
   1. **Method**

For CB/MET complex, 1 mg/ml aqueous solution of MET and 1.21 mg/ml of CB were used after adjustment to pH 8. Different volumes of CB solution were added to 5 ml of MET solution and volume was completed to 10 ml with water (at pH 8). Complexed MET was determined at ʎ_max_= 235 nm. [CB]/[MET] was plotted versus absorbance of complexed MET. The stoichiometric ratio was determined by straight lines extrapolation, and the stability constant (*K_f_*) was determined via **equations (1) and (2)** ^2,3^.

| $\varepsilon b of complexed MET =A/ b CK_{f}= [A/\varepsilon b]/[C_{M}-A/ \varepsilon b] [C_{L}-A/\varepsilon b]$ | (1) | |
| --- | --- | --- |
| $K_{f}= [A/\varepsilon b]/[C_{M}-A/ \varepsilon b] [C_{L}-A/\varepsilon b]\varepsilon b of complexed MET =A/ b$C | | (2) |

Where *εb* is the molar absorptivity constant, *A* is the absorbance at peak point, *b* is cuvette path length = 1 cm, *C* is the molar concentration of complexed MET solution that gives absorbance=*A* at peak point, *C_M_* is the molar concentration of MET, *C_L_* is the molar concentration of ligand (CB or TA) determined from molar ratio at peak point.

For TA/MET complex, 5 ml of MET solution (5 mg/ml, phosphate buffer of pH 5.9) were added to different volumes of TA solution (10.26 mg/ml, phosphate buffer pH 5.9). For each sample, volume was completed to 10 ml and samples were stirred for 10 minutes, then centrifuged at 6000 rpm for 30 minutes. The absorbance of complexed MET was determined indirectly using **equation (3),** then *K_f_* was determined via **equations (1) and (2).**

| $Absorbance of complexed MET \left( \Delta A \right)=A_{MET without TA}-A_{free MET in supernatant}$ | (3) |
| --- | --- |

For SDS/MET, equimolar solutions of MET (0.8281 mg/ml) and SDS (1.4419 mg/ml) prepared in acidified water (pH 2.7) were combined in volumetric ratios ranging from 1:9 to 9:1. The difference in absorbance measured for MET in the absence and presence of ligand is assumed to be proportional to complex concentration. Absorbance of complexed MET of each ratio was measured directly at ʎ_max_=235 nm. SDS mole fraction (X_A_) was plotted versus UV absorbance of complexed drug. Complex stoichiometry and stability constant were determined according to **equation (4)** ^2,3^.

| $K_{f}=(\frac{A}{A_{M}})/\left( \frac{1-A}{A_{M}} \right)^{n+1}C^{n}n^{n}$ | (4) |
| --- | --- |

Where, *A_M_* is maximum absorbance at break point, *A* is actual absorbance, *X* is molar concentration of complexed MET to be determined at this point on the plot, *n* is mole fraction at break point, and *C* is the initial concentration of MET solution used.

- 1. **Results**

Plots obtained for all three complexes are illustrated in **Supplementary Figure 2**.

1. **Statistical analysis**

All experiments were performed at least in triplicate and the results are reported as mean + standard deviation (SD). Statistically significant differences among various treatment groups were assessed utilizing one-way ANOVA (followed by Tukey’s multiple comparisons test) or two-sided Student’s t-test for using GraphPad Prism (version 6.01, GraphPad Software Inc., San Diego, CA).


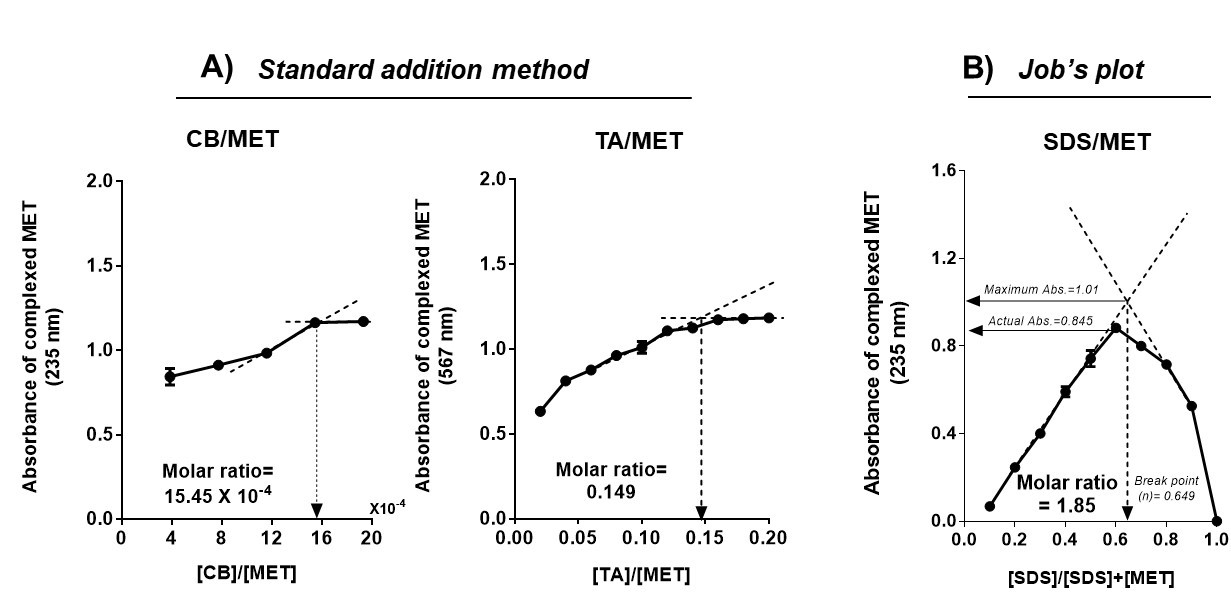


**Supplementary Figure 2: Stability study of different complexes using standard addition method or Job’s plot.** A) Plots of standard addition method for determination of molar ratio of Carbopol/metformin (CB/MET) and tannic acid/MET (TA/MET) complexes. Dotted lines were extrapolated to determine the molar ratio on the x-axis, B) Continuous variation plot (Job’s plot) of sodium dodecyl sulphate/MET (SDS/MET) complex absorbance as a function of SDS molar fraction (Abs.; absorbance). Experiments were performed in triplicate. Data are shown as mean ± SD.

**Supplementary Table 1:** Composition of MET/anionic ligand association complexes.

| Ligand type | Molecular weight | *pK_a_* | Ligand/ MET charge ratio | Ligand/MET molar ratio | Ligand/MET weight ratio |
| --- | --- | --- | --- | --- | --- |
| Carbopol^®^ 940 (CB) | 104,400 | 6 | 1:1 | 7.7 X 10^-4^:1 | 12.1:25 |
| Low methoxy low amidated pectin (LMP) | 59,000 | 3.9 | 1:1 | 4.7 X 10-4:1 | 28: 25 |
| Sodium deoxy cholate (SDC) | 414.6 | 6.5 | 1:1 | 0.33:1 | 20.65: 25 |
| Sodium dodecyl sulphate (SDS) | 288.38 | < 2 | 1:1 < CMC | 2:1 | 4.35: 2.5 |
|  |  |  | 1:1 > CMC | 2:1 | 8.7: 2.5 |
| Tannic acid (TA) | 1,701 | 6 | 1:1 | 0.048:1 | 12.32 :25 |

**Supplementary Table 2:** Partition coefficient of Free MET, CB/ MET and SDS/ MET

| **Complex type** | **Medium pH** | **K_P_ _free MET_** | **K_P complexed MET_** | **Folds increase in lipophilicity** |
| --- | --- | --- | --- | --- |
| CB/MET | 8 | 0.059 + 0.002 | 0.575+ 0.079 | 9.746 + 1.34 |
| SDS/MET | 2.7 | 0.064 + 0.005 | 29.1 + 0.8 | 455.5 + 12.8 |

Data are shown as mean+ SD.

**References**

1 Dominguez, A., Fernandez, A., Gonzalez, N., Iglesias, E. & Montenegro, L. Determination of Critical Micelle Concentration of Some Surfactants by Three Techniques. *Journal of Chemical Education* **74**, 1227-1231, doi:10.1021/ed074p1227 (1997).

2 Syed, A. *et al.* Spectrophotometric Study of Stability Constants of Famotidine-Cu (II) Complex at Different Temperatures. *Journal for Science and Engineering.* **34**, 43-48 (2009).

3 Tirmizi, S. *et al.* Spectrophotometric study of stability constants of cimetidine-Ni(II) complex at different temperatures. *Arabian Journal of Chemistry* **5**, 309-314 (2012).
